# The addition of blood flow restriction during resistance exercise does not increase prolonged low‐frequency force depression

**DOI:** 10.1113/EP091753

**Published:** 2024-04-01

**Authors:** Christopher Pignanelli, Alexa A. Robertson, Steven M. Hirsch, Geoffrey A. Power, Jamie F. Burr

**Affiliations:** ^1^ Department of Human Health & Nutritional Sciences University of Guelph Guelph Ontario Canada; ^2^ Faculty of Kinesiology and Physical Education University of Toronto Toronto Ontario Canada

**Keywords:** ischaemia, oxygen availability, PLFFD, volitional fatigue

## Abstract

At a given exercise intensity, blood flow restriction (BFR) reduces the volume of exercise required to impair post‐exercise neuromuscular function. Compared to traditional exercise, the time course of recovery is less clear. After strenuous exercise, force output assessed with electrical muscle stimulation is impaired to a greater extent at low versus high stimulation frequencies, a condition known as prolonged low‐frequency force depression (PLFFD). It is unclear if BFR increases PLFFD after exercise. This study tested if BFR during exercise increases PLFFD and slows recovery of neuromuscular function compared to regular exercise. Fifteen physically active participants performed six low‐load sets of knee‐extensions across four conditions: resistance exercise to task failure (RE_TF_), resistance exercise to task failure with BFR applied continuously (BFR_CONT_) or intermittently (BFR_INT_), and resistance exercise matched to the lowest exercise volume condition (RE_VM_). Maximal voluntary contraction (MVC) force output, voluntary activation and a force–frequency (1–100 Hz) curve were measured before and 0, 1, 2, 3, 4 and 24 h after exercise. Exercise to task failure caused similar reductions at 0 h for voluntary activation (RE_TF _= 81.0 ± 14.2%, BFR_INT _= 80.9 ± 12.4% and BFR_CONT _= 78.6 ± 10.7%) and MVC force output (RE_TF _= 482 ± 168 N, BFR_INT _= 432 ± 174 N, and BFR_CONT _= 443 ± 196 N), which recovered to baseline values between 4 and 24 h. PLFFD occurred only after RE_TF_ at 1 h supported by a higher frequency to evoke 50% of the force production at 100 Hz (1 h: 17.5 ± 4.4 vs. baseline: 15 ± 4.1 Hz, *P* = 0.0023), BFR_INT_ (15.5 ± 4.0 Hz; *P* = 0.03), and RE_VM_ (14.9 ± 3.1 Hz; *P* = 0.002), with a trend versus BFR_CONT_ (15.7 ± 3.5 Hz; *P* = 0.063). These findings indicate that, in physically active individuals, using BFR during exercise does not impair the recovery of neuromuscular function by 24 h post‐exercise.

## Abstract (highlights)

- **What is the central question of this study?**
  Does the addition of blood flow restriction (BFR) during resistance exercise impaired recovery of neuromuscular function in physically active individuals?
- **What is the main finding and its importance?**
  While BFR during exercise increased fatiguability compared to exercise without BFR, it did not further impair the 24‐h recovery in neuromuscular function compared to exercise without BFR. These findings are relevant for applying BFR exercise in the training and/or rehabilitation of physically active populations such that it does not appear to further exacerbate muscle contractile function compared to regular strenuous exercise.

## 1 INTRODUCTION

Exercise‐induced fatigue includes reversible perceived and performance fatiguability characteristics that impair neuromuscular function (Allen et al., 2008; Enoka & Duchateau, 2016). The intentional reduction of blood flow has long been used to investigate the mechanisms of exercise‐induced fatigue (Gandevia, 2001; Merton, 1954; Reid, 1927); however, only in the past two decades has it gained popularity among researchers and practitioners as a training method (Patterson et al., 2019; Pignanelli et al., 2021). In principle, the intentional limiting of blood flow during exercise, commonly referred to as blood flow restriction (BFR) exercise, reduces or prevents blood flow in and out of the active limbs. At a given load or intensity, the addition of BFR during exercise results in greater motor unit recruitment (Moritani et al., 1992), increased muscle phosphocreatine or glycogen degradation, muscle acidity and muscle lactate accumulation (Greenhaff et al., 1993; Krustrup et al., 2009; Suga et al., 2009, 2010). Metabolite accumulation stimulates type III/IV afferent nerves, which signal the central nervous system, increasing the sensation of pain (Pollak et al., 2014) and decreasing corticospinal excitability (Sidhu et al., 2018). It is likely that this partially explains both the reduction in the time required to reach task failure during BFR exercise (Broxterman, Ade et al., 2015, Broxterman, Craig et al., 2015; Cook et al., 2013; Copithorne & Rice, 2019; Hammer et al., 2020; McClean et al., 2023; Wernbom et al., 2009) and noted impairments immediately after exercise with BFR in common assessments of neuromuscular function such as maximal voluntary contraction (MVC) force output, voluntary activation and twitch force output (Broxterman, Craig et al., 2015; Cook et al., 2013; Hammer et al., 2020; Hill et al., 2022; Husmann et al., 2018; Karabulut et al., 2010; McClean et al., 2023). While these data highlight the immediate effects of BFR, less is known regarding the recovery of neuromuscular function after exercise with BFR. A better understanding of the acute neuromuscular response and recovery is relevant to improving the application of BFR exercise in training and/or rehabilitation.

In the absence of impaired MVC force output, submaximal force output at ‘low’ compared to ‘high’ stimulation frequencies (i.e., ≤20 Hz vs. ≥50 Hz) can be depressed for hours to days, referred to as prolonged low‐frequency force depression (PLFFD; Allen et al., 2008). Since many movements require submaximal force production and motor unit firing rates (e.g., walking, running or cycling), PLFFD may be biologically relevant and related to the increased central motor drive and perception of effort to maintain tasks following exercise (Carson et al., 2002). Investigating the acute neuromuscular adaptations, performance fatigue and PLFFD recovery within hours to days after BFR exercise versus traditional exercise can inform optimal training and rehabilitation strategies, including recovery period optimization. PLFFD may occur due to reduced calcium release from the sarcoplasmic reticulum or myofibrillar calcium sensitivity and is influenced by the exercise mode, recovery period, species and training status (Allen et al., 2008; Bruton et al., 2008; Olsson et al., 2020; Place et al., 2015; Roussel et al., 2023; Skurvydas et al., 2016; Watanabe & Wada, 2016). Reactive oxygen species (ROS) production has been implicated in the development of PLFFD (Andrade et al., 2001; Bruton et al., 2008; Gandra et al., 2018) and a reduction in muscle glycogen may be involved in some (Cheng et al., 2017; Duhamel et al., 2006; Gejl et al., 2014; Nielsen et al., 2014), but not all (Cheng et al., 2020), scenarios. Given that reduced muscle oxygen availability may increase glycogen breakdown during contractions (Greenhaff et al., 1993) and increase ROS production (the latter being controversial; Clanton, 2007), it is conceivable the addition of BFR during exercise may further increase PLFFD compared to regular exercise. Nonetheless, few studies have investigated if BFR increases PLFFD after exercise. In recreationally active individuals, a decreased ratio of 20/50 Hz force output has been reported only immediately after BFR exercise, but not beyond 4 h of recovery (Wernbom et al., 2012). By contrast, a bi‐phasic response was observed for the ratio of 20/100 Hz force output in exercise‐naïve individuals such that the ratio was reduced 1 day and 7 days after the first, but not the second bout of BFR exercise (Sieljacks et al., 2016). This observation coincided with reductions in markers of muscle damage, suggesting a repeated bout effect (Hinks et al., 2021; Sieljacks et al., 2016). An important caveat to these studies was that BFR exercise was performed to task failure, whereas comparisons were made to either volume matched free‐flow exercise, wherein participants stopped at a submaximal number of repetitions done during BFR exercise (Wernbom et al., 2012) or a standardized 150 maximal eccentric muscle actions (Sieljacks et al., 2016). While PLFFD was not a primary outcome of these studies, such comparisons make it difficult to interpret how BFR affects PLFFD compared to a more ecologically valid control, such as a typical free‐flow exercise performed to task failure. Furthermore, comparisons are confounded by the repeated bout effect, which would be expected to occur with exercise‐naïve individuals performing exercise to task failure regardless of BFR. To date, no study has examined if BFR during exercise can impair the recovery in neuromuscular function in the hours to days after exercise while considering total exercise volume performed and perceived effort.

The primary purpose of this study was to test if BFR increases exercise‐induced PLFFD and impairments in common neuromuscular function tests in the recovery period after exercise. We hypothesized that BFR exercise would induce a greater PLFFD and slow the recovery in neuromuscular function compared with exercise without BFR.

## 2 METHODS

### 2.1 Ethical approval

This study was reviewed and approved by the Research Ethics Board at the University of Guelph (REB no. 20‐04‐003) and all participants completed the PARQ+ as well as a general health and physical training questionnaire before providing written informed consent. The study conformed to the standard set by the *Declaration of Helsinki*, except for registration in a database.

### 2.2 Study overview and participant characteristics

**FIGURE 1:**
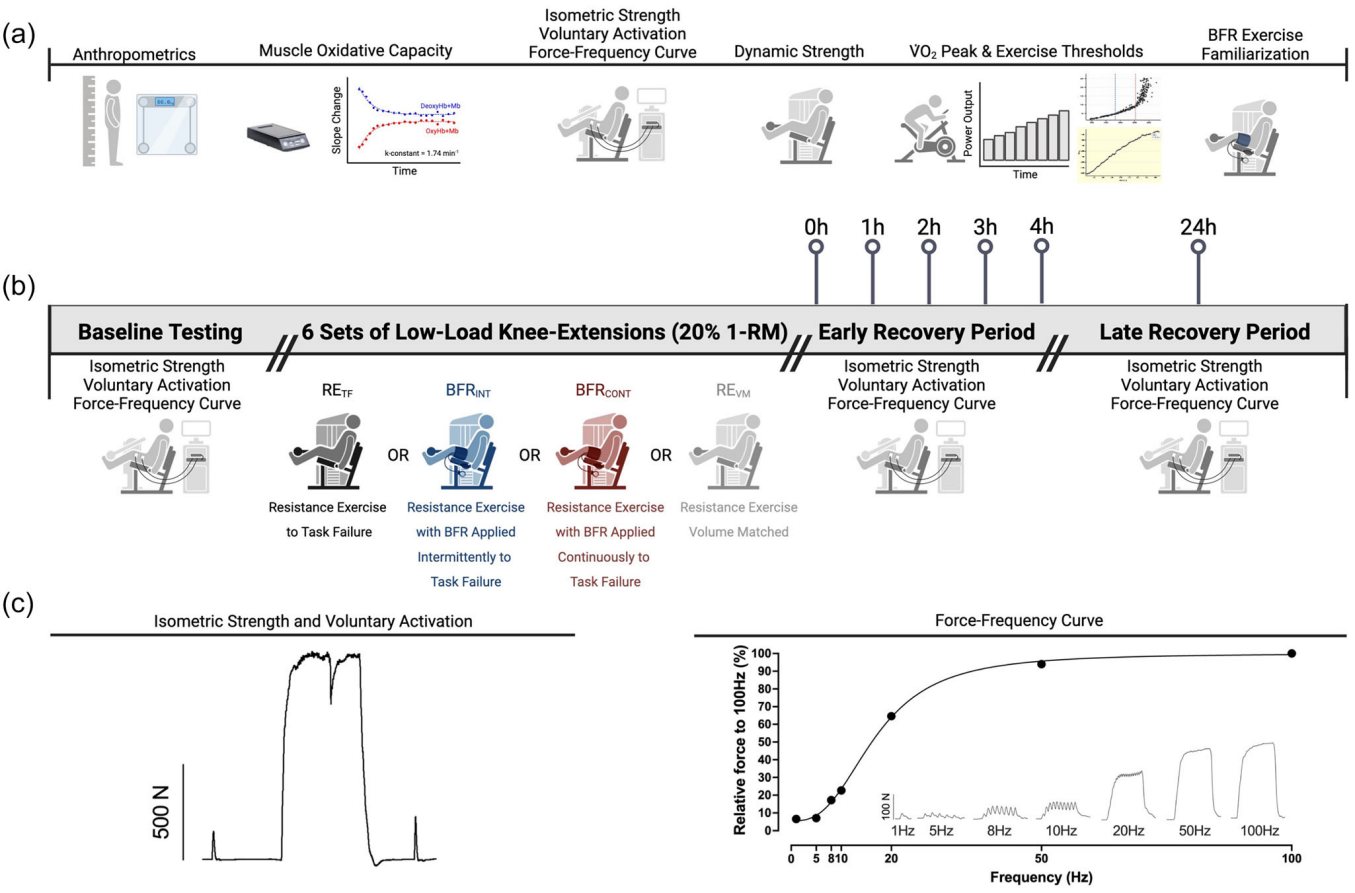
Study overview. (a) Introductory, characterization and blood flow restriction (BFR) familiarization visit. (b) Intervention visit overview with all baseline and post‐exercise neuromuscular testing. Participants performed six sets of knee‐extensions with 20% of their one repetition maximum (1‐RM) under the following conditions: low‐load resistance exercise to task failure (RE_TF_), low‐load resistance exercise to task failure with BFR applied continuously (BFR_CONT_), low‐load resistance exercise to task failure with BFR applied intermittently during exercise sets (BFR_INT_), and low‐load resistance exercise volume matched (RE_VM_) to the condition with the lowest total number of repetitions. (c) Representative tracing of the isometric strength and voluntary activation test (left) as well as the raw force–frequency tracings (right inset). The graph on the right represents the non‐linear regression curve fitting analysis to determine the frequency required to evoke 50% of the force output at 100 Hz. The figure was created using BioRender.com.

Fifteen young, healthy individuals (four females, eleven males) were recruited to participate (Table 1). All participants were engaged in lower body exercise training at least one time per week for 3 months prior to the study. Throughout the study, participants continued their lower body exercise training that included resistance exercise, endurance exercise or both. Participants completed an introductory and familiarization visit, followed by four exercise visits each with a 24‐h follow‐up visit. Each exercise session was spaced 1–2 weeks apart, with participants advised to follow a similar exercise routine the day before every visit. In total, all study visits took approximately 6–8 weeks to complete. The recruitment of only physically active individuals was done so as to have each exercise visit represent a weekly workout as well as to mitigate the contribution of muscle damage and the repeated bout effect, which are expected to be greater in individuals unaccustomed to exercise (Hinks et al., 2021; Sieljacks et al., 2016; Snieckus et al., 2013). An overview of each visit and the measurements obtained within each is presented in Figure 1. Participants arrived at each visit having refrained from exercise and alcohol or recreational drug use (24 h), and caffeine or food (2 h). Participants abstained from pre‐workout supplements, anti‐inflammatory or antioxidant supplements, and recovery modalities such as passive heating or ice baths.

**TABLE 1:** Participant characteristics.

	Female (*n* = 4)	Male (*n* = 11)	Total (*n* = 15)
Age (years)	21.8 ± 1*	25.5 ± 4	24.5 ± 3.6
Height (cm)	163 ± 8*	176 ± 5	173 ± 8
Body mass (kg)	60.4 ± 9.6*	80.0 ± 6.2	74.7 ± 11.3
Body‐fat (%)	22.9 ± 1.8*	14.7 ± 4.4	16.9 ± 5.4
Fat‐free mass (kg)	46.5 ± 7.0*	68.2 ± 5.9	62.4 ± 11.6
Dynamic knee‐extension 1‐RM (kg)	24.3 ± 8.3*	42.7 ± 9.9	37.8 ± 12.5
Peak power			
Absolute (W)	241 ± 57*	340 ± 80	314 ± 86
Relative (W/kg)	3.96 ± 0.36	4.23 ± 0.79	4.16 ± 0.7
Relative (W/FFM kg)	5.14 ± 0.55	4.95 ± 1.18	5.02 ± 0.87
${\dot{V}}_{O_{2}\mathrm{peak}}$			
Absolute (L/min)	2.74 ± 0.63*	4.15 ± 0.60	3.77 ± 0.87
Relative (mL/min/kg)	45.1 ± 3.9*	51.8 ± 5.5	50.0 ± 5.9
Relative (mL/min/FFM kg)	58.6 ± 6.2	61.0 ± 7.6	60.3 ± 7.1
Gas exchange threshold			
Absolute (L/min)	1.48 ± 0.30*	2.02 ± 0.48	1.88 ± 0.49
Relative (mL/min/kg)	24.6 ± 3.2	25.1 ± 4.9	25.0 ± 4.4
Relative (mL/min/FFM kg)	31.9 ± 4.3	29.6 ± 6.3	30.2 ± 5.8
Percentage of ${\dot{V}}_{O_{2}\mathrm{peak}}$	54.7 ± 7.1	48.1 ± 4.9	49.9 ± 6.0
Respiratory compensation point			
Absolute (L/min)	2.22 ± 0.48*	3.22 ± 0.71	2.95 ± 0.79
Relative (mL/min/kg)	36.6 ± 3.1	40.1 ± 7.1	39.2 ± 6.4
Relative (mL/min/FFM kg)	47.5 ± 4.8	47.1 ± 8.5	47.2 ± 7.5
Percentage of ${\dot{V}}_{O_{2}\mathrm{peak}}$	81.2 ± 2.9	77.3 ± 9.4	78.3 ± 8.3
NIRS muscle oxidative capacity *k*‐constant (min^−1^)	2.06 ± 0.66 (*n* = 2)	1.76 ± 0.93 (*n* = 10)	1.81 ± 0.87

*Note*: Data are presented as means ± standard deviation. *P < 0.05 versus males. Abbreviations: 1‐RM, one repetition maximum; FFM, fat‐free mass; NIRS, near‐infrared spectroscopy; ${\dot{V}}_{O_{2}\mathrm{peak}}$, peak oxygen uptake.

### 2.3 Introductory and familiarization visit

Participants sat in a custom‐adapted knee‐extension machine (Element Fitness Carbon Dual 9019 Leg Extension/Leg Curl; The Treadmill Factory, Canada) to test both isometric knee‐extensor force output (via a load cell; Model SML‐300; Durham Instruments, Canada) and dynamic one‐repetition‐maximum (1‐RM). A near‐infrared spectroscopy (NIRS) device was placed on the right leg of the vastus lateralis near the mid‐thigh for the estimation of the quadriceps muscle oxidative capacity. Neuromuscular measures (as described below under ‘Neuromuscular testing’) were then performed to familiarize and assess knee‐extensor function, after which participants performed a dynamic 1‐RM test. In brief, using a robotically controlled resistance machine (1080 Quantum, 1080 Motion, Sweden) the load was increased incrementally after each attempt until participants could no longer perform a full repetition with proper form at the prescribed tempo that was set to a metronome (1.5 s concentric, 1.5 s eccentric). Attempts were separated by 2–3 min. After the dynamic 1‐RM testing, body composition was measured using bioelectrical impedance (BC‐554 Model, Tanita, Japan), and cardiorespiratory fitness was measured using a graded exercise test. Participants with no previous experience performing BFR exercises were asked to perform a familiarization set and rest period (as described under ‘Intervention visits’) with the right leg after all the measurements were completed.

### 2.4 Estimation of muscle oxidative capacity using near‐infrared spectroscopy

Quadriceps oxidative capacity was estimated non‐invasively using a continuous wave NIRS device (PortaMon, Artinis Medical Systems, Netherlands) sampled continuously at 10 Hz with the oxygenated hamoglobin–myoglobin signal calculated using the modified Lambert–Beer law (Barstow, 2019). This test correlates with in vivo magnetic resonance imaging (MRI)‐derived phosphocreatine resynthesis rates (Ryan et al., 2013) and in situ maximal mitochondrial respiration in skeletal muscle (Ryan et al., 2014). This test was used to provide an estimation in quadriceps oxidative capacity of our participants as previous work has shown differences in diseased, untrained (but healthy) and endurance‐trained populations (Adami & Rossiter, 2018). A similar protocol was employed as described in detail elsewhere (Ryan et al., 2012). In brief, two small electrodes above and below the NIRS device were used to electrically stimulate a small portion of the vastus lateralis muscle for 15 s at 5 Hz (pulse width of 200 μs; DS7AH, Digitimer, UK) at a perceived tolerable current intensity controlled through PowerLab 16/35 hardware and recorded on LabChart (Version 8, ADInstruments, Australia). A rapidly inflatable blood pressure cuff system (Hokanson E20 cuff inflator; D.E. Hokanson Inc., USA) was secured as high as possible on the thigh and a belt was wrapped around the cuff. After 3–5 min of rest, there were four cuff inflations (275 mmHg) of 10 s to measure resting oxygen extraction. The muscle was then stimulated to contract for 15 s followed by a series of 20 occlusion–reperfusion cycles of varying length (3–10 s). This procedure was repeated twice more for a total of three tests. A 5–10 min ischaemic calibration was performed after the third test to obtain complete deoxygenated and oxygenated NIRS values. The individual slopes during the occlusion cycles were extracted and analysed offline. Oxygenated haemoglobin–myoglobin values were first corrected for small changes in blood volume during the occlusion using Method 2 (Ryan et al., 2012) and post‐exercise measurements of oxygen extraction were fit to a mono‐exponential curve:

$$y\;\;\left(t\right)=\mathrm{End}-\mathrm{Delta}\times \;e^{-kt}$$

In this equation, *y* represents the relative oxygen extraction during the occlusion, End is the oxygen extraction immediately after the contractions, Delta is the change in oxygen extraction from rest to end of exercise, *t* is time and *k* is the fitting rate constant. It is assumed the recovery rate constant (*k*) of oxygen extraction after exercise is proportional to the muscle oxidative capacity with higher values suggesting greater muscle oxidative capacity and vice versa. Data from two participants could not be obtained due to leg adiposity and one because of a device malfunction.

### 2.5 Cardiorespiratory fitness testing

Cardiorespiratory fitness was assessed using a staged power output test on a cycle ergometer (Velotron, RaceMate, USA). Cardiorespiratory data was collected using the breath‐by‐breath method (Quark CPET, COSMED, Italy). Before each test, the metabolic cart was calibrated with a 3 L fixed‐volume calibration syringe and medical‐grade gas cylinder. The starting power outputs and stage increases were adjusted depending on the estimated aerobic fitness status of the participant: 20 W, +20 W/min for untrained males and females; 50 W, +20 W/min for endurance‐trained females; and 100 W, +30 W/min for endurance‐trained males. Participants cycled until volitional exhaustion and the test was terminated when the cadence decreased by 20–30 rpm despite strong verbal encouragement and/or a clear plateau in ${\dot{V}}_{O_{2}}$ despite increasing the power output. Not all participants displayed a clear plateau, and such data are expressed as a ${\dot{V}}_{O_{2}\mathrm{peak}}$. Raw breath‐by‐breath data were analysed with a freely available online application for exercise thresholds and ${\dot{V}}_{O_{2}\mathrm{peak}}$ values using a 30‐s rolling average (Keir et al., 2022). The ${\dot{V}}_{O_{2}\mathrm{peak}}$ values obtained were compared to the FRIEND database to provide information regarding the cardiorespiratory fitness status of the recruited participants in this study (Kaminsky et al., 2022).

### 2.6 Neuromuscular testing

Participants sat in the same custom‐adapted knee‐extension machine with a load cell (Model SML‐300; Durham Instruments) attached to measure isometric force production. Accounting for thigh girth, two custom made aluminum foil electrode pads were covered in damp paper towels and coated in transmission gel before being affixed on the distal and proximal end of the quadriceps where they were secured with transparent film dressing and tensor wraps to ensure good contact. The right ankle was attached perpendicular to a calibrated load cell by a non‐compliant cuff approximately 2–4 cm above the medial malleolus of the ankle with the knee joint at approximately 90°. All data were acquired through PowerLab 16/35 hardware (ADInstruments) and recorded on LabChart (ADInstruments) sampled at 1000 Hz. The current that elicited the maximal twitch force was found for each participant with a high‐voltage simulator (pulse width of 200 μs; DS7AH) by stimulating participants until the twitch force no longer increased with increasing current. The current used was then increased by 20% for the interpolated twitch technique to estimate voluntary activation (Merton, 1954). A twitch was delivered before, during, and after each MVC attempt and the equation used to calculate voluntary activation is as follows:

$$\begin{array}{ccc} \mathrm{Voluntary}\; & & \mathrm{activation}\;\;\left(\%\right)=\\ & & \left[1-\left(\frac{\mathrm{Change}\;\mathrm{in}\;\mathrm{superimposed}\;\mathrm{twitch}\;\mathrm{force}}{\mathrm{Potentiated}\;\mathrm{twitch}\;\mathrm{force}}\right)\right]\times 100\% \end{array}$$

During each MVC, participants received standardized verbal encouragement as well as visual feedback of previous force production with guidelines displayed to help them attain a higher maximum force value with each attempt. Participants were given a minimum of three attempts separated by 3–5 min of rest. The objective criteria to deem a maximal effort attempt were (1) no further increase in force between attempts and (2) voluntary activation of ≥90%. Participants were given two extra attempts if they believed they could attain a higher force production after the minimum of three attempts. The peak force amplitude was recorded as the MVC. One participant was omitted due to poor or inconsistent voluntary activation values despite similar MVC forces between interventions at baseline. Afterwards, the current was adjusted to elicit 30% of MVC force output when delivered for 1 s at 100 Hz (pulse width of 200 μs) to assess the force–frequency relationship. After 3–5 min of rest, 1 s of electrical stimulation was delivered at the following frequencies, each separated by approximately 20 s: 1, 5, 8, 10, 20, 50 and 100 Hz. Peak force was recorded at each frequency as the highest force output reached during the contraction.

Twitch contraction peak force output and half‐relaxation time were analysed using the Peak Analysis add‐on from LabChart. The half‐relaxation time for 100 Hz tetanic contractions was calculated manually by calculating the time required to decrease force to 50% of the force value at the end of the 100 Hz stimulation. The force–frequency curves were analysed for absolute and relative force production. Relative force production was expressed as a percentage of the force produced at 100 Hz. Non‐linear regression was performed on the relative force production to determine the frequency required to elicit 50% of the force output at 100 Hz (Frequency_50_). The Frequency_50_ value was used to consider the differences day‐to‐day within an individual as well as between individuals for the frequencies at which the muscle summates force. The absolute IC_50_ equation with the least squares regression method was used and the curve fit was constrained at the top and baseline to 100 and 0, respectively (GraphPad Prism software, version 9.5.1, GraphPad Software, Boston, MA, USA). The curve fits throughout the study had *r* ^2^‐values between 0.91 and 0.99.

### 2.7 Intervention visits

Each intervention had two blocks of exercise with three sets in each block for a total of six sets per visit with the prescribed load set to 20% 1‐RM. One minute of rest was given between sets and 5 min of rest was permitted between blocks. Three conditions performed repetitions to task failure in a randomized order: free‐flow low‐load resistance exercise to task failure (RE_TF_), low‐load resistance exercise with BFR applied continuously (BFR_CONT_), and low‐load resistance exercise with BFR applied intermittently (BFR_INT_). The fourth condition was always performed last so that free‐flow low‐load resistance exercise could be matched to the same number of repetitions as the condition that had the least number of repetitions (RE_VM_). The RE_VM_ condition was included since most studies have regular exercise matched to the total time, repetitions or work performed in the BFR exercise condition. Therefore, the inclusion of this condition also allows for inference of our data with previous studies (e.g., Hill et al., 2022; Husmann et al., 2018; Karabulut et al., 2010; Umbel et al., 2009; Wernbom et al., 2012). Task failure was defined as the point where the participant could no longer maintain proper form or tempo for three consecutive repetitions or stopped exercise. The BFR_CONT_ condition had the tourniquet inflated continuously during the exercise set and rest period of each block and was deflated during the 5 min of rest between blocks. The BFR_INT_ condition had the tourniquet inflated only during the exercise sets and was rapidly deflated between sets by the manual removal of the tubing from the tourniquet. The continuous and intermittent application of BFR was used as these are commonly used and advocated (Patterson et al., 2019). The pressure used for the BFR visits was 70% of the lowest effective occlusion pressure, which represents the lowest pressure required to occlude arterial blood flow at rest, as was determined during each visit in a seated position with a tourniquet system (11 cm wide cuff, Personalized Tourniquet System, Delfi Medical Innovations Inc., Canada). The pressure did not differ between BFR visits (BFR_CONT _= 158 ± 20 mmHg and BFR_INT _= 155 ± 19 mmHg). Total exercise volume was calculated by multiplying the total repetitions (number) by the load (kg).

Immediately after the sixth set, participants were equipped with the load cell and neuromuscular testing occurred with the shortest possible delay. Owing to the rapid recovery of MVC force and voluntary activation immediately after exercise, these tests were performed first followed by the force–frequency curve. The tourniquets were deflated during this period, which may underestimate the true impairments in MVC force, twitch contraction properties and voluntary activation, as has been demonstrated previously with ischaemic exercise (Broxterman, Craig et al., 2015; Woods et al., 1987 *b*). All testing immediately after the exercise was completed in approximately 120 s. Neuromuscular testing was performed 1, 2, 3, 4 and 24 h after exercise. Participants remained sedentary in the lab for the first 4 h of recovery and consumed nothing but water. The electrodes remained on the participants throughout the first four hours. Since there are potentiating effects of MVC contractions on low‐frequency force production in the recovery period (Green & Jones, 1989), the force–frequency curve was performed before the MVC and voluntary activation measurements during the 1–4 h recovery period. The 24‐h time point neuromuscular testing was performed as described at baseline.

### 2.8 Statistical analysis

Data were analysed in GraphPad Prism 9 (version 9.5.1) with α set a priori at *P* ≤ 0.05. One‐way ANOVA was used to analyse total repetitions and total exercise volume across the four conditions. Two‐way (4‐Condition by 7‐Time) ANOVA was performed for the remaining neuromuscular outcomes to examine the interaction between each condition and the time course of recovery. When a significant interaction was observed, post‐hoc analysis was performed to examine differences from baseline for each condition with Dunnett's test and for differences between conditions within the same time point with Tukey's test. Data are expressed as means ± standard deviation.

## 3 RESULTS

### 3.1 Participant characteristics and markers of fitness

Participants’ physical characteristics, dynamic knee‐extension 1‐RM and markers of fitness are found in Table 1. Based on reference standards of ${\dot{V}}_{O_{2}\mathrm{peak}}$, females were between the 80th and 90th percentile and males were between the 50th and 90th percentile. The estimate of muscle oxidative capacity (i.e., NIRS *k*‐constant values) falls between healthy and endurance‐trained individuals.

### 3.2 Exercise repetitions and volume

**FIGURE 2:**
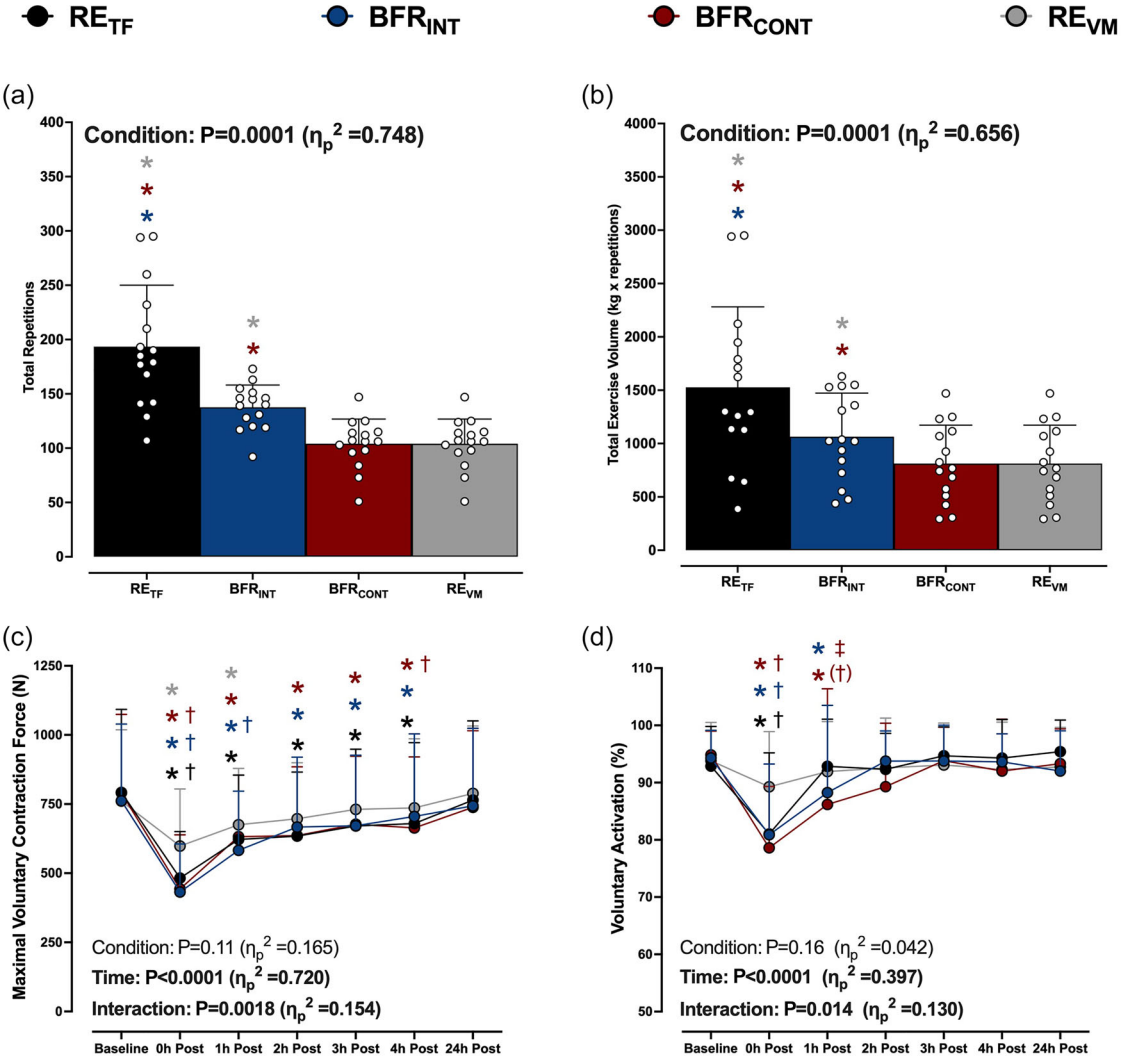
(a, b) Total exercise repetitions (a) and volume (repetitions × load; b) performed after the six sets for low‐load resistance exercise to task failure (RE_TF_; black bars or circles), low‐load resistance exercise to task failure with BFR applied intermittently during exercise sets (BFR_INT_; blue bars or circles), low‐load resistance exercise to task failure with BFR applied continuously (BFR_CONT_; maroon bars or circles), and low‐load resistance exercise volume matched (RE_VM_; grey bars or circles) to the condition with the lowest total number of repetitions. The colour of each asterisk represents the condition that is significantly different from RE_TF_ or BFR_INT_. Data are expressed as means + standard deviation with the white circles representing individual participants (*n* = 15/condition). (c, d) Maximum voluntary contraction force (c) and voluntary activation (d) at baseline and during the 0–24 h recovery period after each condition. ANOVA *P*‐values with partial eta (η_p_^2^) are provided in each panel. Data are expressed as means + standard deviation (*n* = 14/condition). *P < 0.05 versus baseline; †*P *< 0.05 versus RE_VM_ at the same time point; (†)*P* = 0.062 versus RE_VM_ at the same time point; ‡*P *< 0.05 versus RE_TF_ at the same time point. The colour of each symbol represents significantly different conditions. All individual responses can be found in the Supporting information.

The BFR_INT_ and BFR_CONT_ respectively accumulated 25 ± 16% (*P* < 0.0001) and 44 ± 14% (*P* < 0.0001) less exercise volume than RE_TF_. Additionally, BFR_INT_ accumulated 24 ± 14% more exercise volume than BFR_CONT_ and RE_VM_ (both *P* = 0.04; Figure 2a,b).

### 3.3 Maximal force output and voluntary activation

MVC force output decreased from baseline immediately after RE_TF_ (792 ± 300 vs. 482 ± 168N, *P* < 0.0001), BFR_INT_ (762 ± 278 vs. 432 ± 174N, *P* < 0.0001) and BFR_CONT_ (791 ± 284 vs. 443 ± 196N, *P* < 0.0001), all of which were greater reductions (*P ≤* 0.0001) than the reduction after RE_VM_ (761 ± 258 vs. 598 ± 206N, *P* < 0.0001, Figure 2c). MVC force output remained lower for up to 4 h in all conditions, except for RE_VM_, which recovered within 2 h.

Voluntary activation decreased from baseline immediately after RE_TF_ (92.9 ± 6.9 vs. 81.0 ± 14.2%, *P* < 0.0001), BFR_INT_ (94.3 ± 4.8 vs. 80.9 ± 12.4%, *P* < 0.0001) and BFR_CONT_ (94.8 ± 4.2 vs. 78.6 ± 10.7%, *P* < 0.0001), all of which were a greater reduction compared to RE_VM_ (Figure 2d). Voluntary activation remained reduced from baseline 1 h after both BFR_INT_ (88.3 ± 15.2%, *P* = 0.045) and BFR_CONT_ (86.2 ± 20.2%, *P* = 0.001), and only BFR_CONT_ had a lower voluntary activation compared to RE_TF_ (92.9 ± 8.2%, *P* = 0.02), with a trend compared to RE_VM_ (91.9 ± 8.6%, *P* = 0.062) (Figure 2d). All variables returned to baseline values 24 h after exercise.

### 3.4 Twitch and tetanic contractile parameters

**FIGURE 3:**
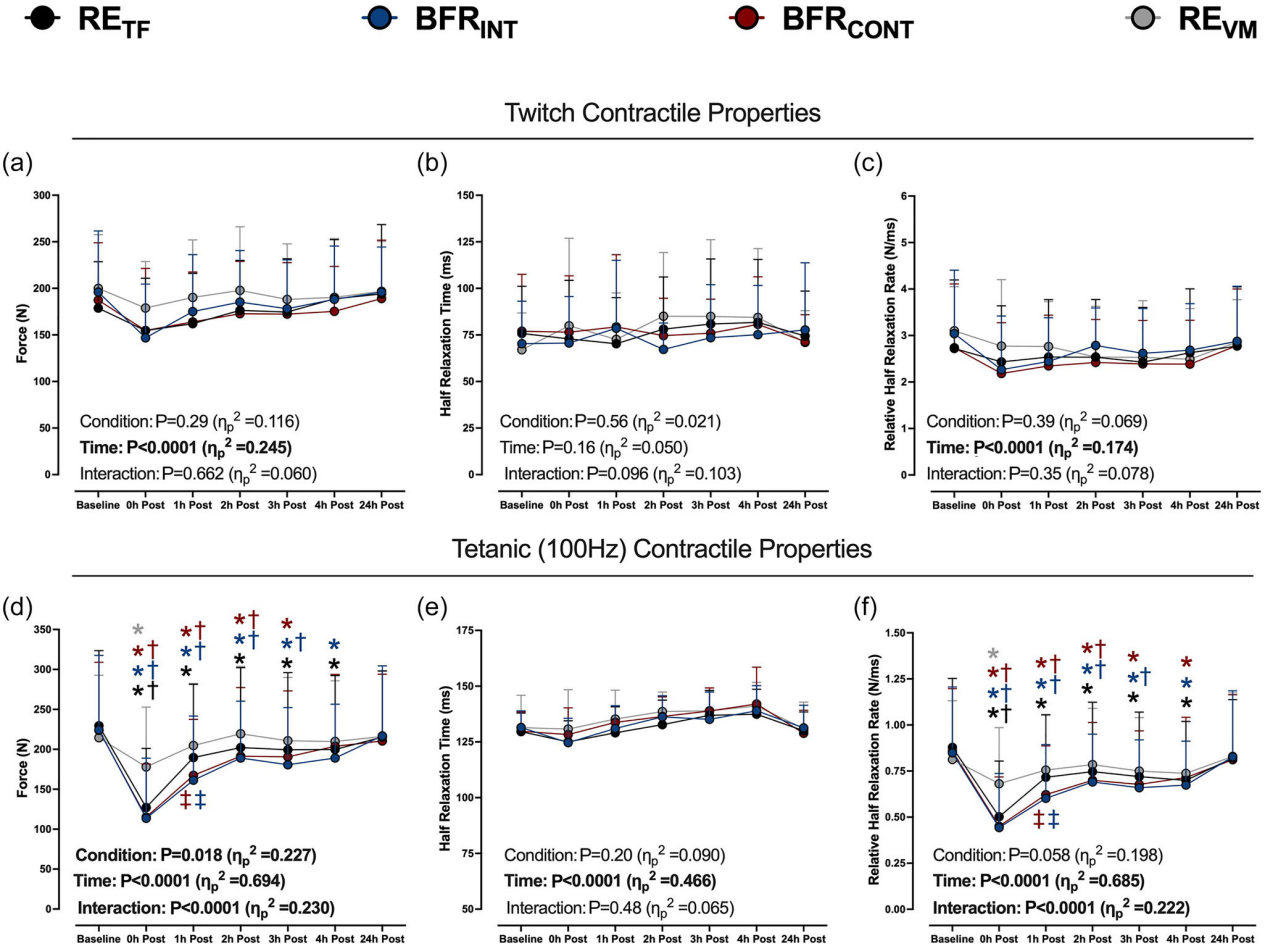
Peak force output, half‐relaxation time, and relative half‐relaxation rate for potentiated twitch contractions (a–c) or tetanic (100 Hz) contractions (d–f) at baseline and 0–24 h after six sets of low‐load resistance exercise to task failure (RE_TF_; black circles), low‐load resistance exercise to task failure with BFR applied intermittently during exercise sets (BFR_INT_; blue circles), low‐load resistance exercise to task failure with BFR applied continuously (BFR_CONT_; maroon circles), and low‐load resistance exercise volume matched (RE_VM_; grey circles) to the condition with the lowest total number of repetitions. ANOVA *P*‐values with partial eta (η_p_^2^) are provided in each panel. *P < 0.05 versus baseline; †*P *< 0.05 versus RE_VM_ at the same time point; ‡*P *< 0.05 versus RE_TF_ at the same time point. The colour of each symbol represents significantly different conditions. Data are expressed as means + standard deviation (*n* = 15/condition). All individual responses can be found in the Supporting information.

No interactions were observed for twitch contraction force output, half relaxation time or relative half relaxation rate (Figure 3a–c). Force output during the 100 Hz contraction was reduced immediately after all exercise, with a significantly greater reduction after RE_TF_, BFR_INT_ and BFR_CONT_ compared to RE_VM_ (Figure 3d). The reduction in force output at 100 Hz persisted for the initial 3 h after exercise only with RE_TF_, BFR_INT_ and BFR_CONT_. No interactions were observed for the 100 Hz half‐relaxation time (Figure 3e). The 100 Hz relative half‐relaxation rates were reduced immediately after each exercise bout and to a greater extent after RE_TF_, BFR_INT_ and BFR_CONT_ compared to RE_VM_ (Figure 3f). The relative half‐relaxation rates were lower from baseline for up to 4 h after RE_TF_, BFR_INT_ and BFR_CONT_. All variables returned to baseline values 24 h after exercise.

### 3.5 Force–frequency relationship

**FIGURE 4:**
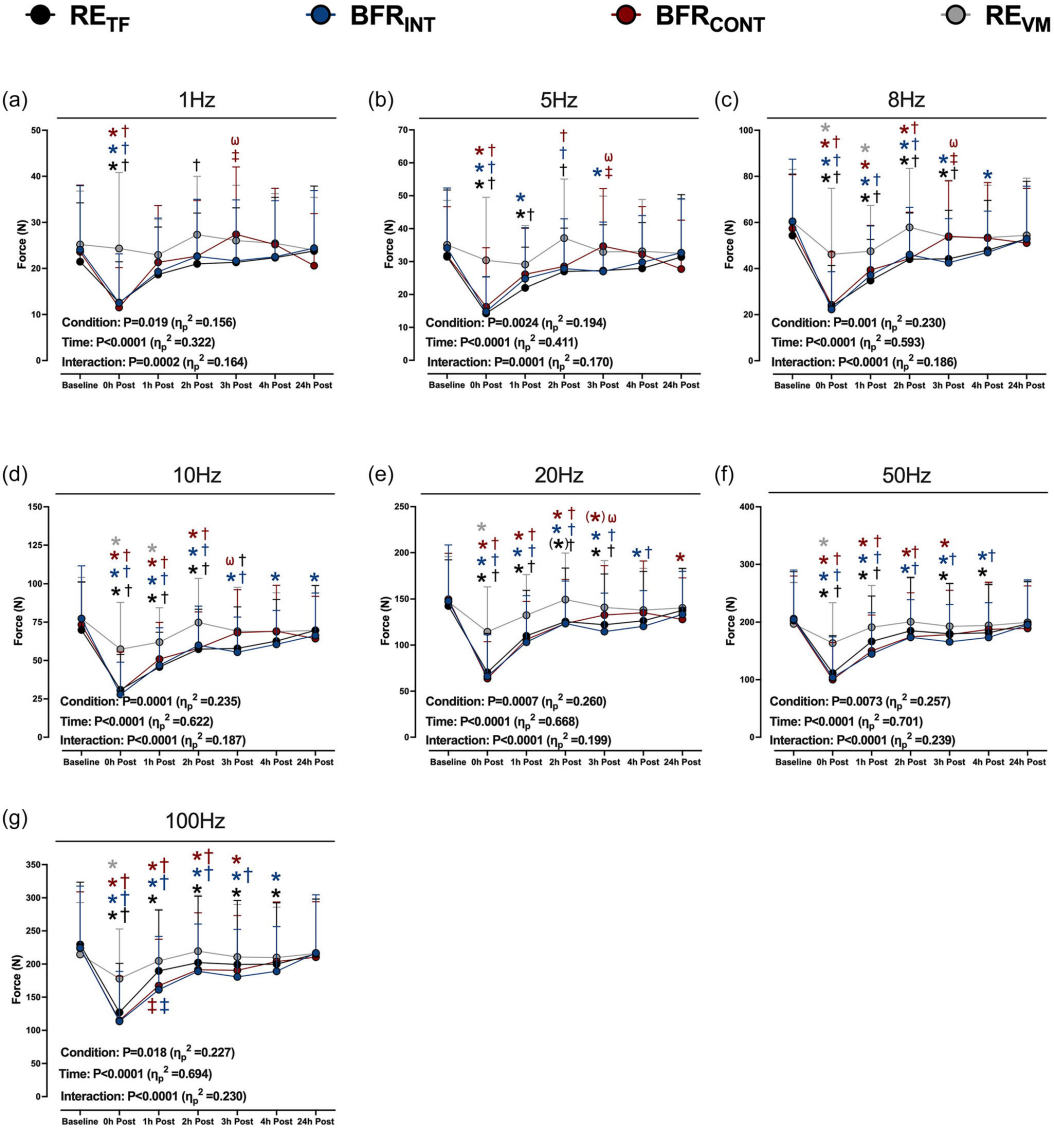
Peak absolute force output at 1 Hz (a), 5 Hz (b), 8 Hz (c), 10 Hz (d), 20 Hz (e), 50 Hz (f), and 100 Hz (g) of stimulation delivered over 1 s at baseline and 0–24 h after six sets of low‐load resistance exercise to task failure (RE_TF_; black circles), low‐load resistance exercise to task failure with BFR applied intermittently during exercise sets (BFR_INT_; blue circles), low‐load resistance exercise to task failure with BFR applied continuously (BFR_CONT_; maroon circles), and low‐load resistance exercise volume matched (RE_VM_; grey circles) to the condition with the lowest total number of repetitions. ANOVA *P*‐values with partial eta (η_p_^2^) are provided in each panel. *P < 0.05 versus baseline; (*)*P* = 0.062 versus baseline; †*P *< 0.05 versus RE_VM_ at the same time point; ‡*P *< 0.05 versus RE_TF_ at the same time point; ω*P *< 0.05 versus BFR_INT_ at the same time point. The colour of each symbol represents a significantly different condition. Data are expressed as means + standard deviation (*n* = 15/condition). All individual responses can be found in the Supporting information.

Force output for the 1–50 Hz contractions was reduced from baseline immediately after exercise in all conditions, except at 1 and 5 Hz for RE_VM_ (Figure 4a–f). The reduction in force output was greater immediately after RE_TF_, BFR_INT_ and BFR_CONT_ compared to RE_VM_. Variable force output recovery across frequencies was observed after exercise; however, the recovery to baseline values after RE_TF_, BFR_INT_ and BFR_CONT_ was generally slower compared to RE_VM_, which recovered toward baseline within 2 h. No differences from baseline were observed 24 h after exercise except for a lower force output at 10 and 20 Hz after BFR_INT_ and BFR_CONT_, respectively.

### 3.6 Prolonged low‐frequency force depression

**FIGURE 5:**
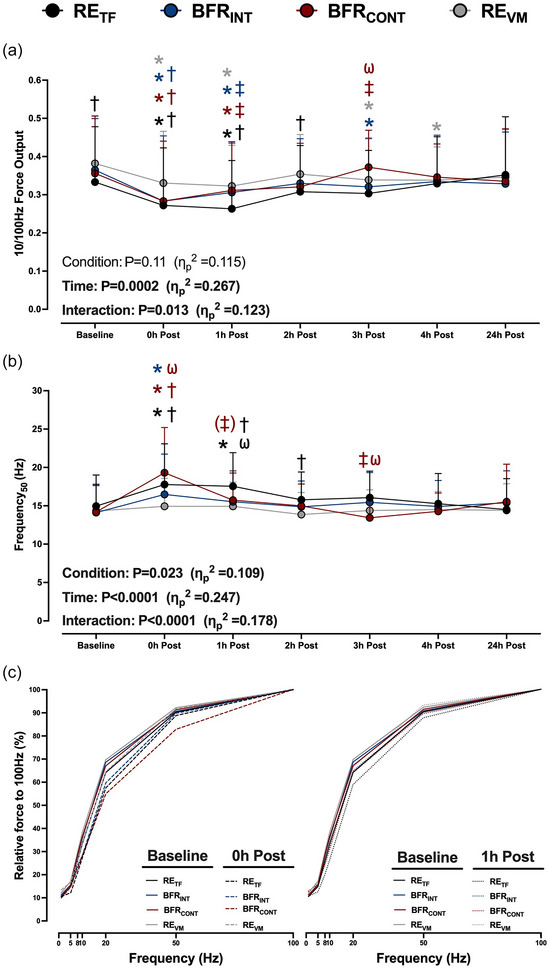
(a, b) The time course recovery of the 10/100 Hz force output (a) and the frequency required to elicit 50% of the force at 100 Hz (Frequency_50_; b) after six sets of low‐load resistance exercise to task failure (RE_TF_; black lines and circles), low‐load resistance exercise to task failure with BFR applied intermittently during exercise sets (BFR_INT_; blue lines and circles), low‐load resistance exercise to task failure with BFR applied continuously (BFR_CONT_; maroon lines and circles), and low‐load resistance exercise volume matched (RE_VM_; grey lines and circles) to the condition with the lowest total number of repetitions. The Frequency_50_ was determined using non‐linear regression analysis. ANOVA *P*‐values with partial eta (η_p_^2^) are provided. (c) Relative force–frequency curves to illustrate the rightward shift 0 h post‐exercise (dashed lines) and 1 h post‐exercise (dotted lines) compared to baseline (continuous lines). Symbols and error bars are omitted for clarity. The mean values and the variance of the relative force output for each frequency can be found in Table 2. *P < 0.05 versus baseline; †*P *< 0.05 versus RE_VM_ at the same time point; ‡*P *< 0.05 versus RE_TF_ at the same time point; (‡)*P* = 0.063 versus RE_TF_ at the same time point; ω*P *< 0.05 versus BFR_INT_ at the same time point. The colour of each symbol represents a significantly different condition. Data are expressed as means + standard deviation (*n* = 15/condition). All individual responses can be found in the Supporting information.

The 10/100 Hz ratio was reduced immediately after all exercise conditions and was lower after RE_TF_, BFR_INT_ and BFR_CONT_ compared to RE_VM_ (Figure 5a). The 10/100 Hz ratio remained lower from baseline 1 h after exercise for each condition with a greater reduction after RE_TF_. The 10/100 Hz ratio recovered to or above baseline after 1 h of recovery for each condition, except for BFR_INT_ (3 h post‐exercise) and RE_VM_ (3 and 4 h post‐exercise).

The Frequency_50_ increased (i.e., a rightward shift in the force–frequency curve) immediately after RE_TF_ (15.0 ± 4.1 vs. 17.8 ± 5.3 Hz, *P* = 0.0007), BFR_INT_ (14.1 ± 3.7 vs, 16.5 ± 5.3 Hz, *P* = 0.006), and BFR_CONT_ (14.2 ± 3.5 vs. 19.3 ± 5.9 Hz, *P* < 0.0001), but not RE_VM_ (14.3 ± 3.3 vs. 14.9 ± 3.6 Hz, *P* = 0.9; Figure 5a). The Frequency_50_ was higher immediately after BFR_CONT_ versus BFR_INT_ (*P* = 0.0007) and RE_VM_ (*P* < 0.0001), whereas the RE_TF_ Frequency_50_ was higher compared to RE_VM_ (*P* = 0.002), but not BFR_CONT_ (*P* = 0.15) or BFR_INT_ (*P* = 0.28). The greater reduction in Frequency_50_ immediately after BFR_CONT_ was primarily due to a greater reduction in relative force production at 50 Hz (Table 2). The Frequency_50_ was only higher from baseline 1 h after RE_TF_ (17.5 ± 4.4 Hz *P* = 0.0023), and was greater than BFR_INT_ (15.5 ± 4.0 Hz, *P* = 0.03) and RE_VM_ (14.9 ± 3.1 Hz, *P* = 0.002) with a trend compared to BFR_CONT_ (15.7 ± 3.5 Hz, *P *= 0.063; Figure 5b). The rightward shift in the force–frequency curves immediately after and 1 h post‐exercise is illustrated in Figure 5c and all relative force output values used to obtain the Frequency_50_ are presented in Table 2.

**TABLE 2:** Relative force–frequency values obtained at low and high‐frequency of electrical muscle stimulation.

	RE_TF_	BFR_INT_	BFR_CONT_	RE_VM_
Baseline				
1 Hz	10.5 ± 6.4	11.2 ± 5.1	11.2 ± 6.9	14.8 ± 8.4
5 Hz	14.8 ± 8.6	15.6 ± 6.7	15.5 ± 8.0	17.1 ± 6.1
8 Hz	25.8 ± 12.8	28.5 ± 11.6	27.9 ± 12.8	29.7 ± 10.6
10 Hz	33.2 ± 14.6	36.3 ± 13.5	35.6 ± 15.0	38.2 ± 12.4
20 Hz	64.2 ± 10.0	67.0 ± 9.5	68.6 ± 9.4	69.7 ± 8.2
50 Hz	90.3 ± 3.2	91.3 ± 4.9	90.7 ± 3.7	92.2 ± 3.0
100 Hz	100 ± 0	100 ± 0	100 ± 0	100 ± 0
0 h post‐exercise				
1 Hz	10.4 ± 6.3	11.1 ± 5.1	9.8 ± 5.2	14.5 ± 6.1
5 Hz	12.1 ± 7.7	15.7 ± 10.7	14.6 ± 10.0	16.6 ± 6.8
8 Hz	21.0 ± 12.0	23.9 ± 17.6	22.7 ± 14.1	25.7 ± 11.2
10 Hz	27.3 ± 15.0*^†^	28.3 ± 16.9*^†^	28.2 ± 15.7*^†^	33.0 ± 13.4*
20 Hz	57.4 ± 14.3*^†^	59.6 ± 12.0*	54.9 ± 13.5*^†^	64.5 ± 8.7
50 Hz	88.8 ± 7.0	89.7 ± 5.3	82.8 ± 13.3*^†ω‡^	91.6 ± 4.0
100 Hz	100 ± 0	100 ± 0	100 ± 0	100 ± 0
1 h post‐exercise				
1 Hz	10.5 ± 5.9	12.7 ± 6.6	12.8 ± 7.0	13.1 ± 5.9
5 Hz	12.5 ± 7.1	16.0 ± 8.8	15.6 ± 7.5	14.8 ± 5.5
8 Hz	20.0 ± 10.2	24.2 ± 11.8	24.2 ± 10.5	24.3 ± 9.2
10 Hz	26.2 ± 12.8*^†^	30.8 ± 12.9*^‡^	31.1 ± 12.8*^‡^	32.1 ± 10.7*
20 Hz	58.9 ± 11.8	64.8 ± 10.6	64.0 ± 11.2^‡^	66.3 ± 9.5
50 Hz	87.8 ± 4.3	90.0 ± 5.4	89.6 ± 5.2	93.2 ± 3.7^‡^
100 Hz	100 ± 0	100 ± 0	100 ± 0	100 ± 0
2 h post‐exercise				
1 Hz	11.5 ± 6.2	12.1 ± 5.6	12.1 ± 5.7	15 ± 6.6
5 Hz	14.8 ± 7.1	15.1 ± 7.3	15.1 ± 6.3	17.2 ± 6.8
8 Hz	23.9 ± 9.3^†^	25.2 ± 10.1	24.0 ± 9.4	27.1 ± 9.6
10 Hz	30.9 ± 12.1^†^	32.9 ± 11.7	32.0 ± 11.3	35.5 ± 10.5
20 Hz	63.6 ± 11.3	66.2 ± 9.7	66.2 ± 8.4	70.0 ± 9.1
50 Hz	91.4 ± 3.8	91.9 ± 4.0	91.5 ± 3.3	91.9 ± 3.4
100 Hz	100 ± 0	100 ± 0	100 ± 0	100 ± 0
3 h post‐exercise				
1 Hz	11.2 ± 5.7	11.9 ± 5.8	14.6 ± 5.1*	15.6 ± 7.8
5 Hz	14.3 ± 6.1	15.0 ± 7.5	18.1 ± 5.8	15.4 ± 6.0
8 Hz	23.4 ± 9.2	24.2 ± 10.1	29.1 ± 8.3	25.8 ± 9.0
10 Hz	30.4 ± 11.3	32.0 ± 12.8*	37.2 ± 9.6^‡ω^	33.7 ± 10.8*^‡^
20 Hz	62.1 ± 9.7	64.9 ± 12.3	71.5 ± 10.2^ω‡^	67.9 ± 7.6
50 Hz	89.9 ± 4.2	91.8 ± 4.6	94.1 ± 4.6	91.2 ± 3.7
100 Hz	100 ± 0	100 ± 0	100 ± 0	100 ± 0
4 h post‐exercise				
1 Hz	11.8 ± 6.4	12.0 ± 5.3	12.6 ± 4.6	14.2 ± 5.9
5 Hz	14.7 ± 7.1	16.0 ± 7.0	16.2 ± 5.5	15.7 ± 5.7
8 Hz	25.4 ± 10.7	26.1 ± 10.2	26.7 ± 7.9	25.8 ± 7.8
10 Hz	32.9 ± 12.1	33.4 ± 12.0	34.6 ± 8.7	33.7 ± 8.7*
20 Hz	65.0 ± 11.9	65.2 ± 9.8	67.2 ± 6.4	67.0 ± 7.2
50 Hz	91.0 ± 5.5	92.1 ± 2.8	92.3 ± 3.9	92.3 ± 2.8
100 Hz	100 ± 0	100 ± 0	100 ± 0	100 ± 0
24 h post‐exercise				
1 Hz	11.7 ± 7.0	11.8 ± 5.5	11.4 ± 7.1	13.9 ± 5.8
5 Hz	15.6 ± 9.3	15.8 ± 7.4	14.7 ± 8.0	15.6 ± 6.2
8 Hz	26.8 ± 13.2	26.2 ± 11.4	26.8 ± 11.9	26.7 ± 10.3
10 Hz	35.2 ± 15.3	32.9 ± 13.6	33.5 ± 13.7	34.5 ± 12.4
20 Hz	65.9 ± 11.0	63.3 ± 10.2	63.6 ± 14.1	67.4 ± 10.2
50 Hz	91.7 ± 3.1	90.2 ± 3.9	90.1 ± 6.7	93.5 ± 4.5
100 Hz	100 ± 0	100 ± 0	100 ± 0	100 ± 0

*Note*: Relative force production at low and high frequencies at baseline and 0–24 h after six sets of low‐load resistance exercise to task failure (RE_TF_), low‐load resistance exercise to task failure with BFR applied intermittently during exercise sets (BFR_INT_), low‐load resistance exercise to task failure with BFR applied continuously (BFR_CONT_), and low‐load resistance exercise volume matched (RE_VM_) to the condition with the lowest total number of repetitions. Data are expressed as a percentage of relative force produced at 100 Hz for each time point. *P < 0.05 versus baseline; †*P *< 0.05 versus RE_VM_ at the same time point; ‡*P *< 0.05 versus RE_TF_ at the same time point; ω*P *< 0.05 versus BFR_INT_ at the same time point. Data are expressed as means ± standard deviation (*n* = 15/condition).

## 4 DISCUSSION

The primary aim of this study was to examine if BFR during exercise increases PLFFD and slows the recovery of neuromuscular function compared to regular exercise. The data indicate that PLFFD is not further increased after BFR exercise on the first day of recovery. However, relative force output during high‐frequency electrical muscle stimulation was reduced immediately after exercise only when BFR was applied continuously. Notably, BFR exercise does not cause prolonged recovery impairments in neuromuscular function compared to regular exercise in physically active individuals.

### 4.1 MVC force output, voluntary activation and twitch parameters as markers of neuromuscular function recovery after exercise with and without BFR

The current data support that both intermittent and continuous BFR application during exercise reduce the amount of time required to cause immediate impairments in common tests of neuromuscular function (i.e., MVC force output, voluntary activation, twitch force). This is reinforced by evidence of greater impairments in neuromuscular function after BFR_CONT_ compared to RE_VM_, which is the commonly used comparative condition (Hill et al., 2022; Husmann et al., 2018; Karabulut et al., 2010; Umbel et al., 2009; Wernbom et al., 2012). However, similar impairments were observed immediately after exercise for all conditions that performed exercise to task failure with each condition performing a different number of repetitions (RE_TF _> BFR_INT _> BFR_CONT_). Our data align with Cook et al. (2013), who observed similar impairments in neuromuscular function immediately after exercise to task failure with and without BFR; however, they are in contrast to others showing greater impairments after BFR exercise to task failure (Broxterman, Craig et al., 2015). A possible explanation is that Broxterman and colleagues assessed function while the tourniquets remained inflated (Broxterman, Craig et al., 2015). Since neuromuscular function remains impaired while the tourniquet is applied and recovers within 3 min of intact circulation (Woods et al., 1987), our data and Cook et al. (2013) may have underestimated the impairment in neuromuscular function immediately after BFR exercise. Despite these discrepancies immediately after exercise, MVC force output, voluntary activation, and the twitch parameters recovered back to baseline between 2 and 24 h after exercise with or without BFR. While a recent commentary has highlighted the potential influence of BFR during exercise to induce muscle damage (Wernbom et al., 2020), our data indicate this to be less likely in individuals of greater overall fitness as supported by ${\dot{V}}_{O_{2}\mathrm{peak}}$ values in the 50–90th percentile (Kaminsky et al., 2022) and NIRS‐derived estimates of muscle oxidative capacity placing participants between healthy and endurance‐trained (Adami & Rossiter, 2018). The recovery in MVC force output (an indirect marker of exercise‐induced muscle damage; Damas et al., 2016) supports this notion as the relative values at 24 h were on average 92%–99% of baseline values regardless of whether exercise was performed with BFR.

### 4.2 Influence of BFR on exercise‐induced PLFFD

Our findings indicate PLFFD was not further induced with BFR exercise compared to regular exercise. If anything, BFR may have attenuated PLFFD as RE_TF_ had a greater reduction in the 10/100 Hz ratio and an increased Frequency_50_ value from baseline 1 h after exercise, a time point where exercise metabolites that impair force production (e.g., inorganic phosphate; Debold et al., 2016) are likely near resting levels. Since PLFFD is attenuated with a mitochondrial‐specific antioxidant in rodents (Gandra et al., 2018) and mitochondrial ROS emission is reduced 2 h after BFR resistance exercise (Petrick et al., 2019), it is possible PLFFD was only induced after RE_TF_ due to an attenuated mitochondrial ROS production after BFR exercise. Alternatively, it could be due to the addition of BFR during exercise reducing force output at high frequencies of stimulation. The absolute force output at 100 Hz was reduced to a greater extent with both BFR exercise conditions compared to RE_TF_, but with similar reductions at low frequencies 1 h after exercise (Figure 4). A lower 100 Hz force output with similar low‐frequency force output results in higher relative force output at the lower frequencies of stimulation (Table 2) and subsequently lower Frequency_50_ values (Figure 5). Collectively, our data do not provide strong evidence that BFR during exercise increases PLFFD compared to regular strenuous exercise.

Although most data presented here imply the method of BFR application (i.e., intermittently vs. continuously) did not greatly affect the neuromuscular function recovery and PLFFD, we provide novel evidence to suggest the relative force output at 50 Hz was reduced only immediately after BFR_CONT_. Since the relative 50 Hz force output recovered to baseline within 1‐h post‐exercise, this supports a transient change that occurred only when BFR was applied continuously. Indeed, previous work has shown muscle inorganic phosphate is increased with BFR exercise when applied continuously versus intermittently (Suga et al., 2012) and muscle excitability is reduced during ischaemic contractions in young individuals (Chung et al., 2007), both of which reduce force output at high‐frequency electrical stimulations (Dahlstedt et al., 2001; Jones et al., 1979). While we cannot confirm these possibilities with the current data, future work could examine why this was observed and its potential relevance beyond a single exercise bout.

### 4.3 Methodological considerations

There are several considerations worth noting. First, the entire study took at most 8 weeks to complete. This study design was intentional to improve ecological validity by mimicking a weekly training session in physically active individuals. Nonetheless, it is possible cardiorespiratory fitness levels or strength may have changed throughout the study. Cardiorespiratory fitness was not tracked in this study; however, similar MVC force outputs were observed at the first and last exercise intervention (786 ± 292 vs. 760 ± 260 N, *P* = 0.8), supporting limited changes in maximal strength. Second, the crossover design may have resulted in a repeated bout effect that could affect the interpretation of the data (Hinks et al., 2021; Sieljacks et al., 2016). However, the repeated bout effect is minimized after a third bout in individuals unaccustomed to exercise (Chen et al., 2009). Since we recruited and randomized physically active individuals who were performing regularly training before and throughout the study, the potential repeated bout effect is likely minimal. Lastly, due to the nature of the interventions, neither the participant nor researcher was blinded to testing allocation; however, the likelihood of participant expectation affecting the outcome measures is low.

### 4.4 Conclusion

The use of BFR during exercise has attracted the interest of researchers, practitioners, and athletes to facilitate training adaptations. A better understanding of how the neuromuscular system recovers in the hours to days after exercise with BFR is needed to tailor its application to training and/or rehabilitation. We indicate BFR reduced the time required to impair neuromuscular function immediately after exercise (e.g., MVC force output, voluntary activation, and twitch parameters). However, the recovery time course following BFR exercise is similar to non‐BFR exercise.

## AUTHOR CONTRIBUTIONS

Conception and study design: Christopher Pignanelli, Geoffrey A. Power and Jamie F. Burr Data collection, and analysis: Christopher Pignanelli, Alexa A. Robertson and Steven M. Hirsch Initial manuscript draft: Christopher Pignanelli Manuscript interpretation, and revision: Christopher Pignanelli, Alexa A. Robertson, Steven M. Hirsch, Geoffrey A. Power and Jamie F. Burr All authors have read and approved the final version of this manuscript and agree to be accountable for all aspects of the work in ensuring that questions related to the accuracy or integrity of any part of the work are appropriately investigated and resolved. All persons designated as authors qualify for authorship, and all those who qualify for authorship are listed.

## CONFLICT OF INTEREST

The authors declare no conflicts of interest.

## Supporting information

Supplementary material ‐ Individual responses

## Data Availability

Data will be made available upon request.
