# Supplementary material for: The addition of blood flow restriction during resistance exercise does not increase prolonged low‐frequency force depression
Source: Exp Physiol. 2024 Apr 1;109(5):738–53. doi: 10.1113/EP091753 (PMC11061635; doi:10.1113/EP091753)
Supplement: Supplementary file 1 — Supplementary material ‐ Individual responses [file EPH-109-738-s001.docx]

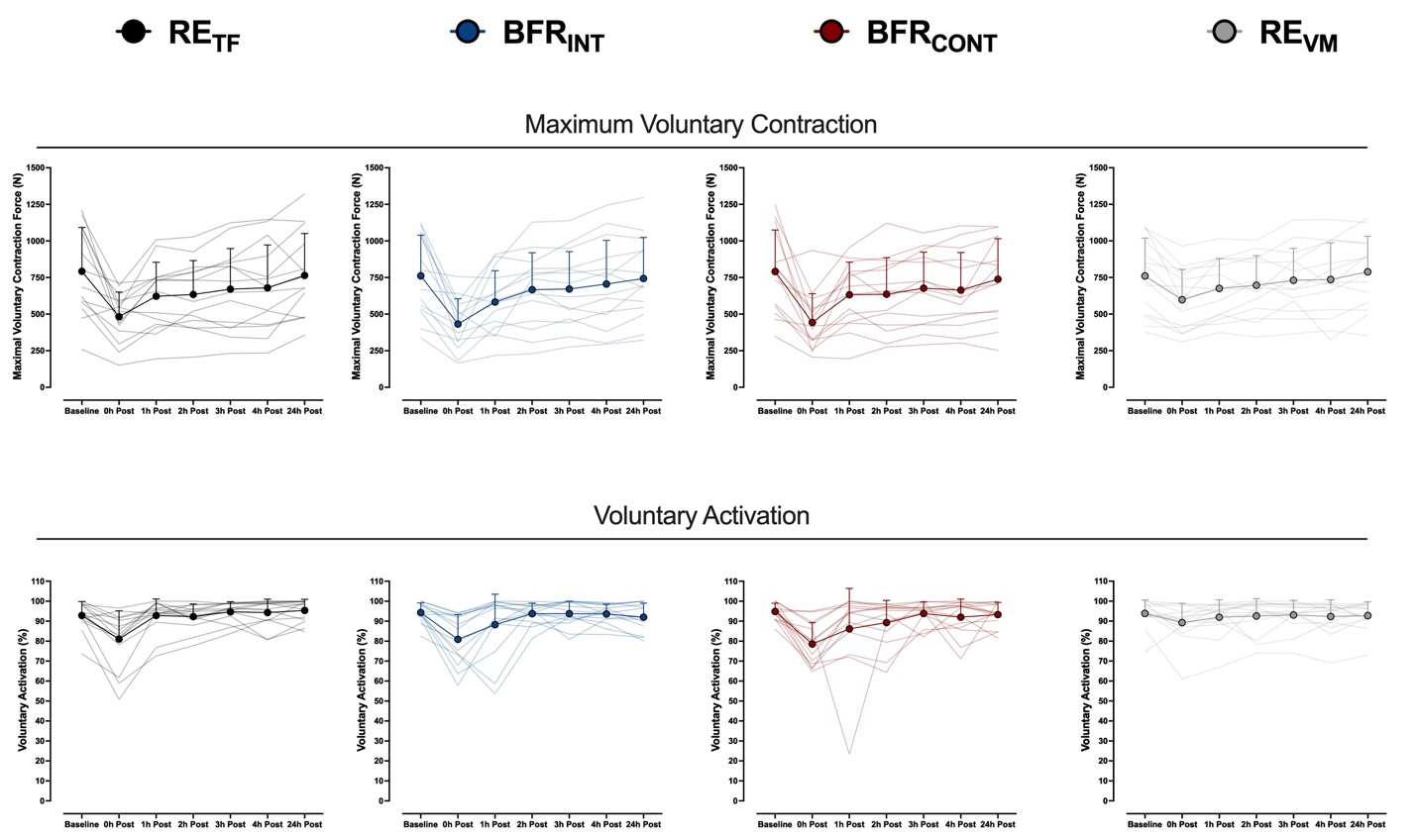


**Figure 2.** Individual responses for maximum voluntary contraction force output and voluntary activation.


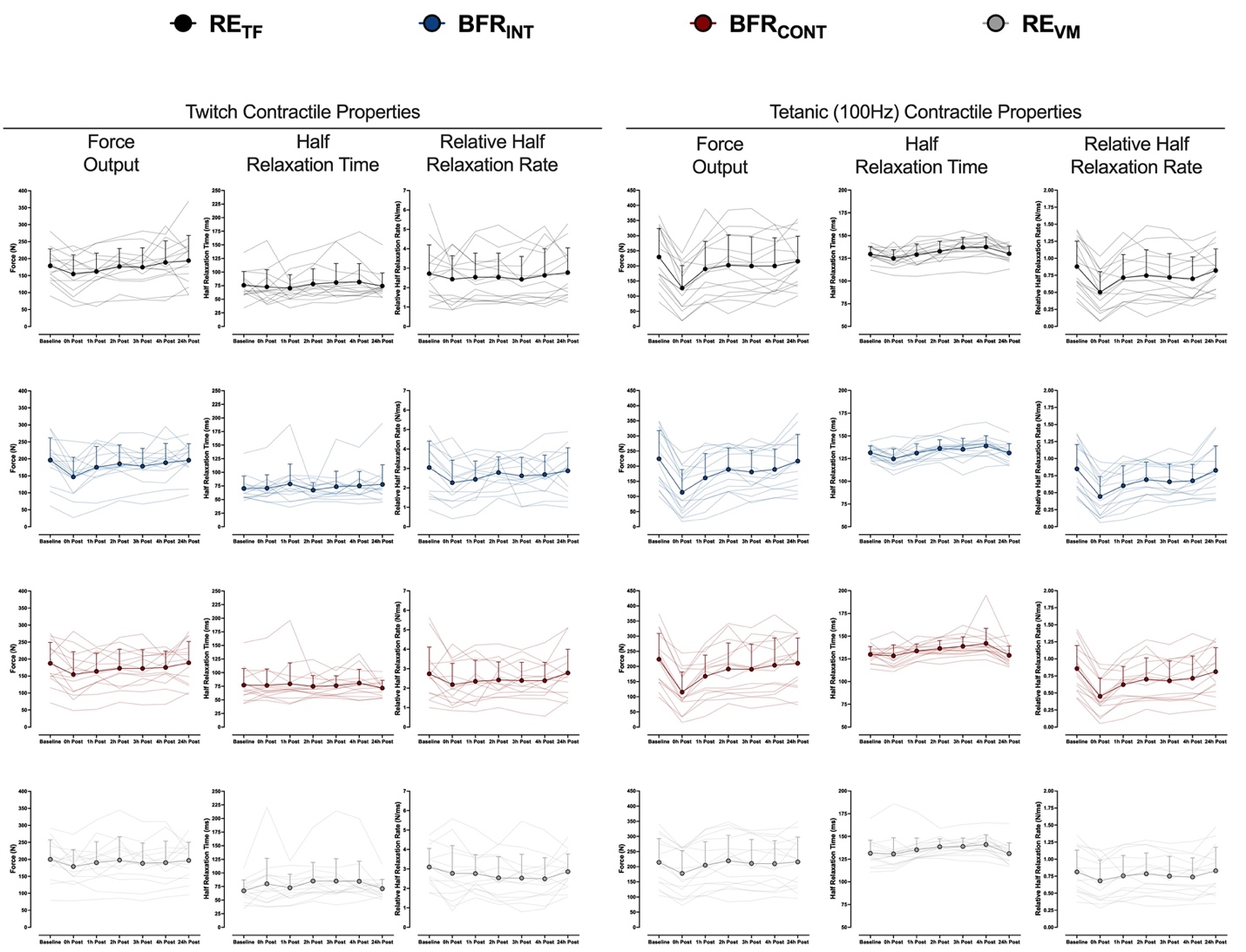


**Figure 3.** Individual responses for twitch and tetanic (100Hz) peak force output, half relaxation time, and relative half relaxation rate.

**Figure 4.** Individual responses for peak force output at 1, 5, 8, 10, 20, and 50Hz.


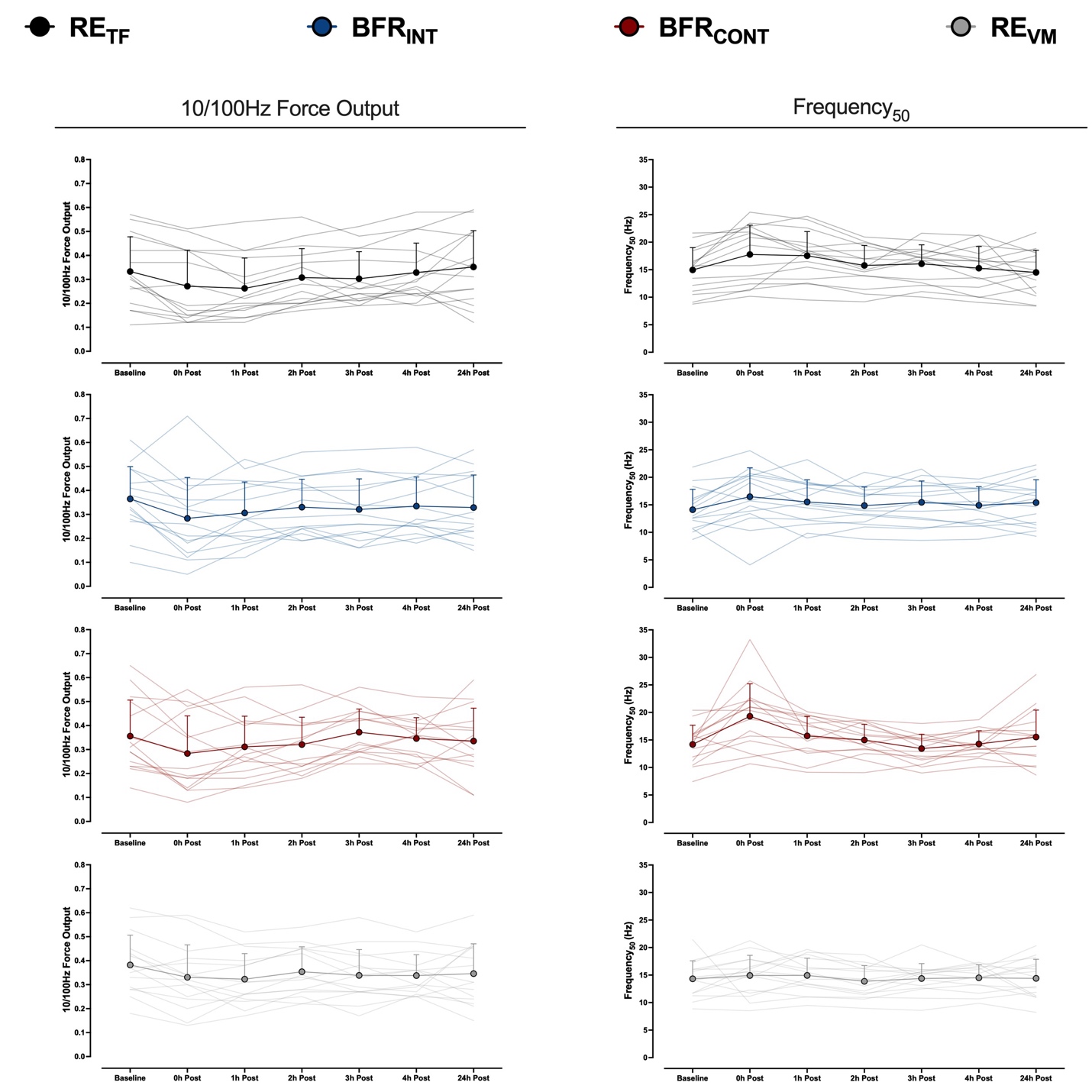


**Figure 5.** Individual responses for 10/100Hz relative force output and the Frequency_50_.
